# Immuno-oncology safety education experience: Key lessons from ipilimumab (IPI)

**DOI:** 10.1186/2051-1426-3-S2-P384

**Published:** 2015-11-04

**Authors:** Heddy Bartell, Jedd Wolchok, F Stephen Hodi, Helen Liu, Cynthia Wojtaszek, Jeffrey Weber

**Affiliations:** 1 Bristol-Myers Squibb, Plainsboro, NJ, USA; 2 Memorial Sloan Kettering Cancer Center, New York, NY, USA; 3 Dana-Farber Cancer Institute, Boston, MA, USA; 4 Bristol-Myers Squibb, Princeton, NJ, USA; 5 Bristol-Myers Squibb, jackson, NJ, USA; 6 H. Lee Moffitt Cancer Center, Tampa, FL, USA

## Background

IPI (Yervoy^®^, Bristol-Myers Squibb) blocks cytotoxic T-lymphocyte antigen-4, a regulatory molecule on activated T cells. In March 2011, IPI, indicated for unresectable or metastatic melanoma (MEL), became the first immune checkpoint inhibitor approved by the US Food and Drug Administration (FDA). Early in development, severe and fatal immune-related adverse events (irAEs) emerged and were attributed to IPI's mechanism of action, occurring mostly during treatment, although a minority occurred weeks to months after drug discontinuation. irAE management guidelines created in collaboration with external experts and study investigators were progressively incorporated into the IPI Investigator's Brochure.

## Methods

At approval, the FDA stipulated that a Risk Evaluation and Mitigation Strategy (REMS) communication plan directed at healthcare providers (HCPs) be developed to ensure that benefits with IPI outweighed irAE risks. The REMS goal was to inform HCPs about serious risks with IPI and irAE management. Multichannel educational outreach to HCPs was implemented at launch and every 6 months for 3 years, in addition to a dedicated webpage and triggered outreach to new prescribers. REMS materials included a Dear HCP Letter, management guide, nursing checklist, and patient (pt) wallet card. Audience was HCPs who manage pts with MEL (eg, oncologists) and HCPs who may be consulted about irAEs (eg, gastroenterologists). Professional medical societies were also contacted to inform their memberships.

## Results

During the 3-year outreach effort, REMS materials were distributed via >160,000–250,000 emails per outreach and >700,000 total mailings. Prescriber understanding of irAE management was assessed twice, via oncologist knowledge surveys, with median correct scores of 76% in 2012 and 85% in 2014. Academic and community HCPs tested equally well. Pharmacovigilance review of fatal irAEs showed no delay in recognizing and treating irAEs, and management was consistent with US prescribing information. Periodic, aggregate safety data did not identify any new safety concerns. Because the REMS met its goal and key activities were successfully completed with no required modifications at the 18-month and the 3-year assessments, the FDA released the REMS in March 2015. Voluntary IPI safety education efforts continue worldwide.

## Conclusions

Management algorithms and safety education materials were created through early collaboration with investigators and then broadly disseminated through the IPI REMS, increasing HCP awareness of irAE management. Socialization of irAE identification and management through the sentinel IPI safety education experience has benefited newer immuno-oncology agents, and IPI safety materials remain highly regarded and utilized by HCPs.

